# SILAC-based quantification of changes in protein tyrosine phosphorylation induced by Interleukin-2 (IL-2) and IL-15 in T-lymphocytes

**DOI:** 10.1016/j.dib.2015.08.007

**Published:** 2015-08-22

**Authors:** Nerea Osinalde, Virginia Sánchez-Quiles, Vyacheslav Akimov, Blagoy Blagoev, Irina Kratchmarova

**Affiliations:** Department of Biochemistry and Molecular Biology, University of Southern Denmark, Odense M, Denmark

**Keywords:** SILAC, Interleukin, Cell signaling, Phosphotyrosine, T-lymphocytes

## Abstract

This data article presents the first large-scale quantitative phosphoproteomics dataset generated to decipher the signaling networks initiated by IL-2 and IL-15 in T-lymphocytes. Data was collected by combining immunoprecipitation of tyrosine phosphorylated proteins and TiO_2_-based phosphopeptide enrichment with SILAC-based quantitative mass spectrometry. We report all the proteins and phosphotyrosine-containing peptides identified and quantified in IL-2- and IL-15-stimulated T-lymphocytes. The gene ontology analysis of IL-2 and IL-15 effector proteins detected in the present work is also included. The data supplied in this article is related to the research work entitled “Simultaneous dissection and comparison of IL-2 and IL-15 signaling pathways by global quantitative phosphoproteomics” [1]. All mass spectrometry data have been deposited in the ProteomeXchange with the identifier PXD001129.

**Specifications Table**

**Table:** 

Subject area	Cell signaling and immunology
More specific subject area	Interleukin signaling and quantitative phosphoproteomics
Type of data	Mass spectrometry (MS) data
How data was acquired	MS data was acquired in a Q-Exactive (Thermo) mass spectrometer.
Data format	Raw (^⁎^raw), excel files (.xlsx)
Experimental factors	Kit225 T-cells were grown in light (Arg0/Lys0), medium (Arg6/Lys4) and heavy (Arg10/Lys8) media. Differentially SILAC-labeled T-cells were kept unstimulated, treated with IL-2 or stimulated with IL-15, respectively prior cell lysis.
Experimental features	After stimulation, cells were lysed and protein extracts derived from the three different experimental conditions were combined and enriched in tyrosine phosphorylated proteins using two antibodies. Immune complexes were fractionated in a SDS-PAGE and in-gel digested using trypsin. Resulting peptides were either directly analyzed by LC-MS/MS or enriched in phosphorylated peptides using TiO_2_ beads prior MS analysis.
Data source location	Odense, Denmark
Data accessibility	All MS data presented in this article are deposited in the ProteomeXchange with the identifier PXD001129 (http://proteomecentral.proteomexchange.org/datastet/PXD001129).
List of all proteins identified in each of the two replicas performed are provided in Supplementary material linked with this article.

**Value of the data**

• The first simultaneous dissection of the signaling pathways triggered by IL-2 and IL-15 in CD4+ T-cells provides extensive data that allows discerning between the proteins that are regulated or not by tyrosine phosphorylation upon cytokine stimulation.
• The detection of numerous cytokine-dependent and – independent tyrosine phosphorylation events enables constructing a more precise molecular snapshot of the ongoing events on T-cells treated with IL-2 and IL-15.
• The identification of previously not reported phosphorylated tyrosine residues corresponding to distinct proteins serves as the starting point to characterize their biological relevance.

## 1 Data, experimental design, materials and methods

In the present work we provide the data generated to unveil the signaling pathways initiated by IL-2 and IL-15 in T-lymphocytes [1]. We include two tables containing quantitative and qualitative information about all the proteins and phosphotyrosine (pY)-containing peptides identified and quantified in the pY-immune complexes isolated from IL-2- and IL-15-treated T-lymphocytes, as well as the gene ontology analysis was performed.

**Fig. 1:**
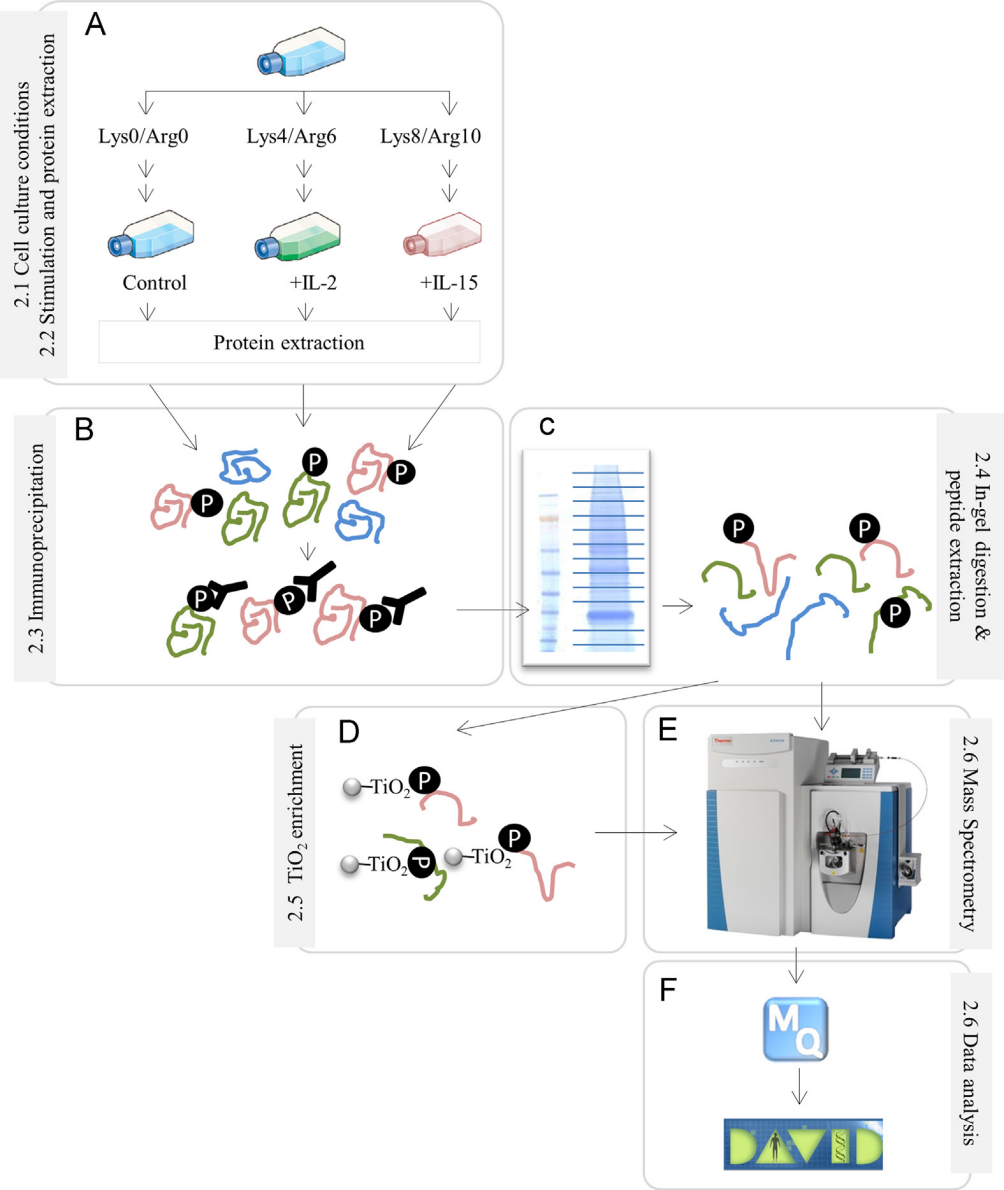
Experimental design of the SILAC-based quantitative proteomics/phosphoproteomics experiment.

To assess the signaling networks initiated downstream of IL-2/IL-2R and IL-15/IL-15R complexes in T-lymphocytes, we followed the experimental workflow shown in Fig. 1. (A) Kit225 T-cells were grown in media containing either light (0/0), medium (6/4) or heavy (10/8) version of arginine and lysine until their proteome was completely labeled. Then, cells grown in light media were kept unstimulated and thus served as control whereas cells grown in medium and heavy media were stimulated with IL-2 and IL-15, respectively. After protein extraction, differentially labeled cell lysates were combined in 1:1:1 ratio. (B) Tyrosine phosphorylated proteins were enriched using phospho-specific antibodies and (C) fractionated on a SDS-PAGE. After protein in-gel digestion and peptide extraction, resulting peptides where either (D) enriched in phosphopeptides using TiO_2_ beads prior mass spectrometry (MS) analysis or (E) directly analyzed by a QExactive mass spectrometer. (F) Acquired raw mass spectra data were analyzed using the MaxQuant software and further data analysis was performed using David bioinformatics tools.

## 2 Materials and methods

### 2.1 Cell culture conditions

The human T-cell chronic lymphocytic leukaemia-derived, IL-2 dependent Kit225 cell line [2] was maintained in RPMI 1640 medium supplemented with 10% FBS, 1% Glutamax, 1% penicillin/streptomycin (p/s), 1% sodium pyruvate and 16 U/ml of recombinant human IL-2 (kindly provided by AIDS Research and Reference Reagent Program, Division of AIDS, NIAD, NIH, USA) at a density of 1×10^6^ cells/ml.

For SILAC experiments, Kit225 T-cells were grown in custom-made RPMI deficient for L-Arg, and L-Lys (Gibco-Invitrogen, Carlsbad, CA), which was supplemented with 10% dyalized serum (Gibco-Invitrogen), 1% Glutamax, 1% p/s, 1% sodium pyruvate and distinct isotopes of L-Arginine (18 µg/ml) and L-Lysine (730 µg/ml). Externally added amino acids included: L-Arginine (Arg0), L-Lysine (Lys0), L-Arginine-^13^C_6_ (Arg6), L-Lysine-^2^H_4_ (Lys4), L-Arginine-^13^C_6_^15^N_4_ (Arg10) and L-Lysine-^13^C_6_^15^N_2_ (Lys8) from Sigma-Aldrich. Thus, Kit225 T-cells were grown either in light (Arg0/Lys0), medium (Arg6/Lys4) or heavy (Arg10/Lys8) media for at least five divisions prior cytokine stimulation to allow complete labeling of their proteome.

### 2.2 Cell stimulation and protein extraction

Kit225 T-cells were IL-2-deprived for 48 h in order to arrest the cells at the G1 phase of the cell cycle and resemble resting T-cells before cytokine treatment. Signaling cascades were induced by adding 200 U of IL-2 or IL-15 (later one purchased from PeproTech) to Kit225 cells and incubation for a period of 5 min at 37 °C.

For SILAC experiments Kit225 cells grown in light media (Arg0/Lys0) were kept unstimulated whereas cells grown in medium (Arg6/Lys4) and heavy media (Arg10/Lys8) were stimulated with IL-2 and IL-15, respectively, as described above.

Treatment was quenched by keeping cells on ice for 5 min and rapidly proceeding with cell lysis using ice-cold modified RIPA buffer (50 mM Tris–HCl pH 7.5, 150 mM NaCl, 1% NP-40, 1 mM EDTA, 0.25% sodium deoxycholate, 1 mM sodium pervanadate, 5 mM beta-glycerophosphate, 5 mM NaF, complete protease inhibitor cocktail (Complete tablets, Roche)). Proteins were recovered from the supernatant of the samples after 15 min centrifugation at 13,000 rpm, 4 °C. Finally, protein concentration was determined with the BCA assay (Pierce).

### 2.3 Immunoprecipitation of tyrosine phosphorylated proteins

The three differentially labeled protein lysates derived from untreated, IL-2-stimulated and IL-15-stimulated T-cells were combined in 1:1:1 ratio according to their protein concentration and incubated with 50% slurry protein A-sepharose (GE Healthcare) for 1 h at 4 °C to eliminate proteins that bind non-specifically to the beads. Pre-cleared protein lysates were then incubated with two complementary anti-phosphotyrosine antibodies; for 3 h at 4 °C with agarose conjugated 4G10 (Millipore, MA, USA) and for additional 3 h with immobilized p-Tyr-100 antibody (Cell Signaling, Beverly, MA). Eluted immune complexes were equally split and run on 2 parallel lanes of a precast gradient NuPAGE 4–12% Bis-Tris Gel (Invitrogen) and visualized with Colloidal Blue (Invitrogen) (Supplementary Fig. 1).

### 2.4 In-gel digestion and peptide extraction

Gel lanes were cut into slices and subjected to in-gel reduction, alkylation and trypsin digestion basically as previously described [3]. Briefly, gel slices were discolored and dehydrated by serial incubations with 100 mM ammonium bicarbonate/100% ethanol (1/1, vol/vol) and 100% ethanol. Peptide reduction was achieved by incubating gel slices with 10 mM DTT for 45 min at 56 °C, followed by dehydration and peptide alkylation which was performed by incubation with 55 mM chloroacetamide for 30 min at room temperature in the dark. Subsequently, gel slices were incubated with 100% ethanol until they became white and shrink to finally perform the protein digestion by adding trypsin (12.5 ng/µl) and incubating overnight at 37 °C. To keep gel pieces wet during enzymatic cleavage add 30–50 µl of ammonium bicarbonate buffer on top of the gels.

Peptides were extracted from the gel by serial 10 min incubations with 100% acetonitrile (ACN) and 30% ACN/3% TFA at room temperature in a shaker. The supernatant obtained in the distinct incubations were pooled and dried down in a vacuum centrifuge.

### 2.5 Enrichment of phosphorylated peptides using TiO_2_ beads

Enrichment of phosphopeptides was performed as previously described [4]. Titansphere beads (5 µm diameter) were mixed with the equilibration buffer (50 mg/ml DHB in 80% ACN/1% TFA) in 1:1 (w/w) for 30 min at room temperature and samples were adjusted to 60% ACN/1% TFA. 1–2 µl of equilibrated TiO_2_ slurry was added to the sample and incubated for 30 min at room temperature with rotation. After brief centrifugation, supernatant was removed and TiO_2_ beads were transferred onto a C_8_ STAGE Tip where they were washed two times is 60% ACN/1% TFA and then eluted with 2×15 µl 5% NH_4_OH followed by 2×15 µl 25% NH_4_OH/0.3% TFA.

### 2.6 Mass spectrometry analysis

Prior MS analysis all samples were desalted and concentrated on a C_18_ STAGE-tip. Peptides were eluted with 50% ACN/1% TFA, dried down in a vacuum centrifuge and finally resuspend in 2% ACN/0.3% TFA. Acidified peptide mixtures were separated on a 75-μm inner diameter 20-cm-long column packed in-house with ReproSil-Pur C18-AQ 3 μm resin (Dr. Maisch GmbH). Reverse-phase chromatography was performed with an EASY-nLC 1000 ultra-high pressure system (Thermo Fisher Scientific), which was coupled to the Q Exactive mass spectrometer (Thermo Fisher Scientific) via a nanoelectrospray source (Thermo Fisher Scientific). Peptides were loaded in solvent A (0.5% acetic acid) and eluted with a nonlinear 140-min gradient of 8–45% solvent B (0.5% acetic acid, 80% ACN) at a flow rate of 250 nl/min. MS instrument was operated to automatically switch between full-scan MS (*m*/*z* range, 300–1750; maximum injection time 120 ms; resolution 70,000 at *m*/*z* 400; target of 10^6^ ions) and up to 10 data-dependent MS/MS scans (maximum injection time 124 ms; resolution 35,000 at *m*/*z* 400; target of 10^4^ ions). Precursors were fragmented by higher-energy C-trap dissociation (HCD) with normalized collision energy of 25 eV. Repeat sequencing of peptides was minimized by excluding the selected peptide candidates for 45 s.

### 2.7 Data analysis

Acquired raw mass spectra data from both biological replicates were combined and processed using the MaxQuant software v1.3.0.5. applying the parameters as previously described [5]. For protein identification at least two peptides, including 1 unique were required. Both razor and unique peptides, except Phospho-STY modified peptides, were considered for protein group quantification. Supplementary Table 1 contains information about all identified and quantified proteins.

**Fig. 2:**
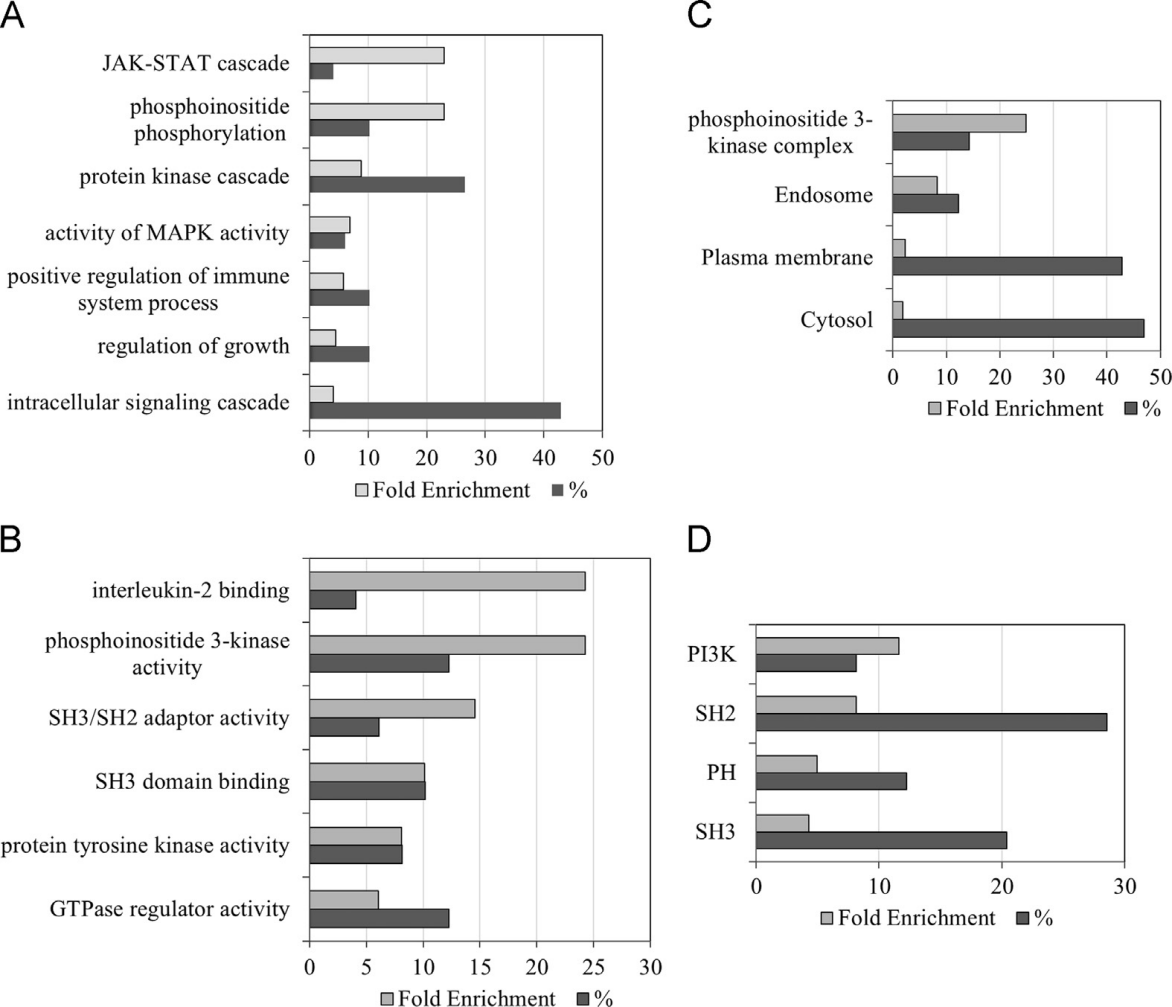
Gene ontology analysis of proteins enriched in phosphotyrosine immunocomplexes isolated upon 5 min stimulation with IL-2 and IL-15. The most representative terms corresponding to (A) biological process, (B) molecular function, (C) cellular compartment and (D) protein domains that we found enriched within our dataset of IL-2 and IL-15 effector proteins are represented by fold enrichment of the terms as well as by % of proteins within our dataset included in such a term.

The biological functions, cellular localization and proteins domains overrepresented among the proteins enriched (IL-2/Ctr and/or IL-15/Ctr ratio>1.5 and statistically significance value *p*<0.01) in the phosphotyrosine immunocomplexes upon cytokine stimulation were studied using The Database for Annotation, Visualization and Integrated Discovery (DAVID) [6]. Within our dataset of IL-2 and IL-15 effector proteins we detected that several (Fig. 2A) biological processes, (Fig. 2B) molecular functions, (Fig. 2C) cellular compartments and (Fig. 2D) protein domains were highly enriched.

For the analysis of pY-containing peptides, 1% FDR, a minimum localization probability of 0.75 and score difference of 5 (ClassI phosphopeptides) were demanded [7,8]. Supplementary Table 2 contains information about all identified and quantified pY-containing ClassI and ClassII phosphopeptides.
