# Supplementary figures and images for: SILAC-based quantification of changes in protein tyrosine phosphorylation induced by Interleukin-2 (IL-2) and IL-15 in T-lymphocytes
Source: Data Brief. 2015 Aug 22;5:53–8. doi: 10.1016/j.dib.2015.08.007 (PMC4564383; doi:10.1016/j.dib.2015.08.007)

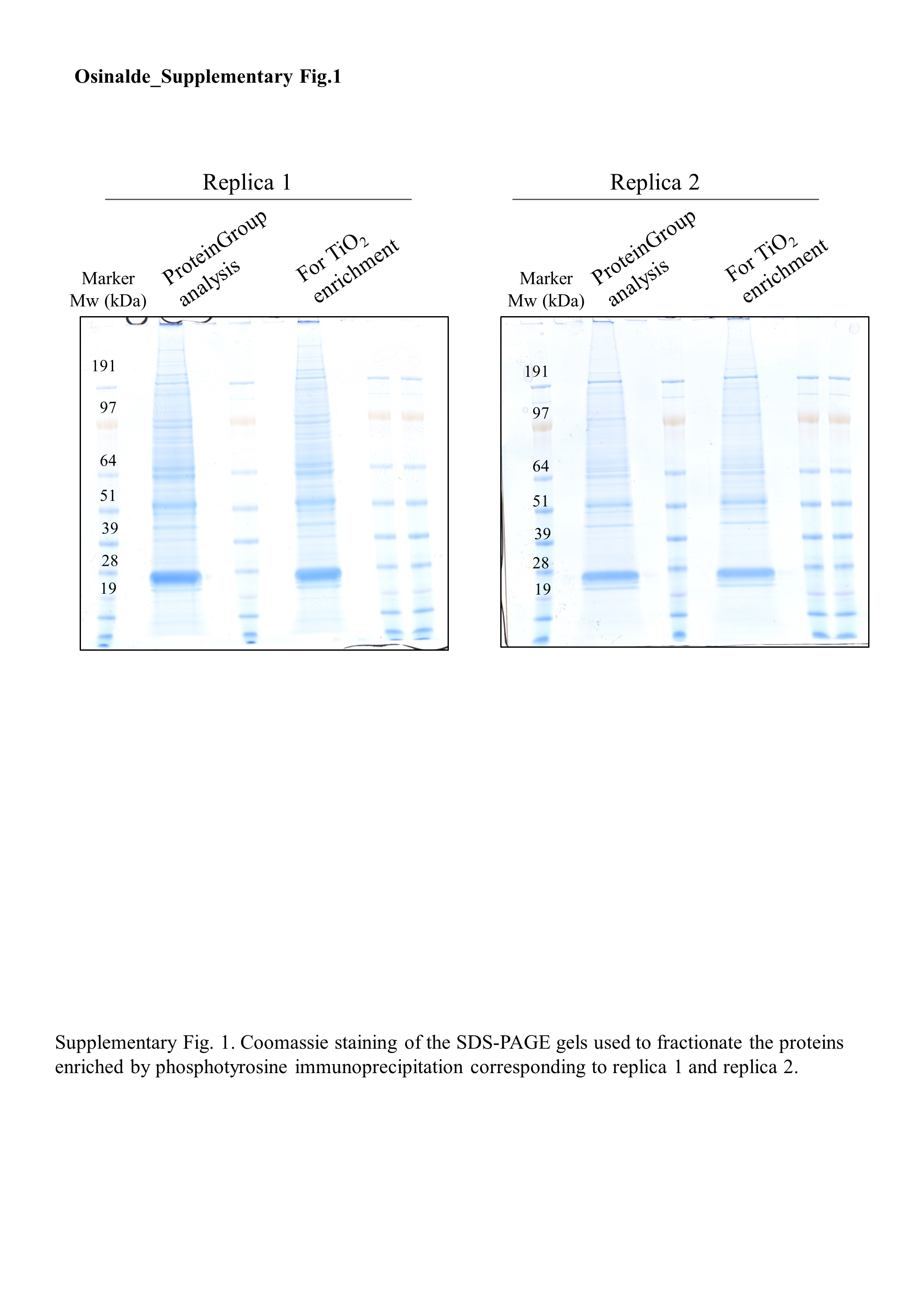

Supplement: Supplementary file 1 — Supplementary data [file mmc1.zip › Suppl. Fig. 1.tif]
