# Supplementary material for: SILAC-based quantification of changes in protein tyrosine phosphorylation induced by Interleukin-2 (IL-2) and IL-15 in T-lymphocytes
Source: Data Brief. 2015 Aug 22;5:53–8. doi: 10.1016/j.dib.2015.08.007 (PMC4564383; doi:10.1016/j.dib.2015.08.007)
Supplement: Supplementary file 4 — Supplementary data [file mmc4.doc]

10 August 2015

Dear Members of the editorial board of Data in Brief journal,

Hereby, the authors of manuscript DIB-D-15-00153 entitled “SILAC-based quantification of changes in protein tyrosine phosphorylation induced by Interleukin-2 (IL-2) and IL-15 in T-lymphocytes” declare no conflict of interest.

With best regards,

Irina Kratchmarova
